# ﻿Biodiversity restated: > 99.9% of global species in Soil Biota

**DOI:** 10.3897/zookeys.1224.131153

**Published:** 2025-02-03

**Authors:** Robert J. Blakemore

**Affiliations:** 1 VermEcology, 101 Suidomichi, Nogeyama, Yokohama-shi, Kanagawa-ken 231-0064, Japan VermEcology Kanagawa-ken Japan; 2 ENSSER, Marienstr. 19/20, 10117 Berlin, Germany ENSSER Berlin Germany; 3 IUCN Species Survival Commission, Rue Mauverney 28, 1196 Gland, Switzerland IUCN Species Survival Commission Gland Swaziland

**Keywords:** Bacteria, earthworm, microbes, -Omics, soil organisms, species richness, viruses

## Abstract

More than a decade of research led to the conclusion in 2022 that the Soil Biome is home to ~ 2.1 × 10^24^ taxa and thus supports > 99.9% of global species biodiversity, mostly Bacteria or other microbes, based upon topographic field data. A subsequent 2023 report tabulated a central value of just 1.04 × 10^10^ taxa claiming soils had 59 ± 15%, i.e., 44–74% (or truly 10–50%?) of the global total, while incidentally confirming upper values of ~ 90% for soil Bacteria. Incompatibility of these two studies is reviewed, supporting prior biodiversity data with the vast majority of species inhabiting soils, despite excluding viruses (now with ~ 5 × 10^31^ virions and 10^26^ species most, ~ 80%, in soils). The status of Oligochaeta (earthworms) and other taxa marked “?” in the 2023 paper are clarified. Although biota totals are increased considerably, inordinate threats of topsoil erosion and poisoning yet pertain with finality of extinction. Species affected include Keystone taxa, especially earthworms and microbes, essential for a healthy Soil foundation to sustain the Tree-of-Life inhabiting the Earth.

## ﻿Introduction

**Figure 1.**
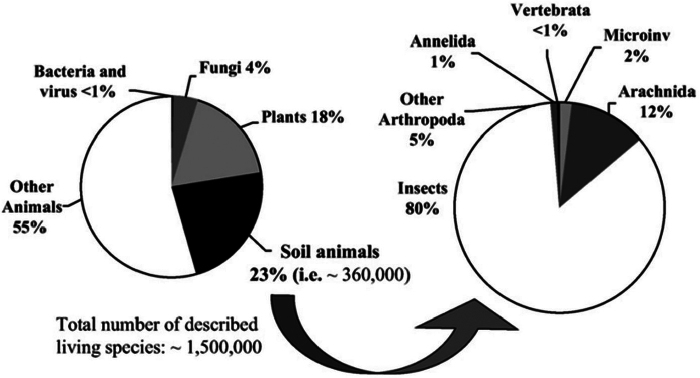
Decaëns et al. (2006: fig. 1) had “soil animals 23%” (i.e., ~ 360,000 in ~ 1.5 million species = 24%?).

Healthy soil is fundamental to sustainable existence of most species evolving on Earth in Darwin’s “Tree of Life” (a paradigm defended by Gulik et al. 2024). Soil supports more than 99.9% of species diversity and, now that vascular plants that seed and root in soils are included (Blakemore 2024), it supports 99% biomass hence ~ 98% of Net Primary Productivity (NPP) and also O_2_ production. Bar-On and Milo (2019) had 0.7 Gt of photosynthetic/oxygenase Rubisco enzyme powering terrestrial environments (doubled for terrain to 1.4 Gt) with just ≈ 0.03 Gt (2.1%) in the marine environment. Soil filters and stores freshwater stocks (being subject to Earth tides!) and, as well as ~ 99% of human food, it provides most building materials plus many of our essential medicines/antibiotics. Thus, an important metric must be the scope and snapshot status of living or dormant Soil biota. A recent review by Anthony et al. (2023) claimed “two times greater soil biodiversity than previous estimates”, seemingly because Decaëns et al. (2006) had 23% “soil animals” in their tally of described species as known at that time (Fig. 1). Both assertions are challenged for several reasons, not least > 90–99% Soil biota reports by Williamson et al. (2017), Bickel and Or (2020), Zhao et al. (2022), and by Blakemore (2018b, 2022, 2023).

Much higher totals had been determined since 2006, and Williamson et al. (2017) concluded: “Soils represent the greatest reservoir of biodiversity on the planet; prokaryotic diversity in soils is estimated to be three orders of magnitude greater than in all other ecosystems combined.” In other words, soils may contain 99.9% of species, mainly microbes. Supporting this were, for example, Bickel and Or (2020) or Zhao et al. (2022) who said: “soil is the most microbiologically abundant (10^29^) and diverse (10^11^) environment on the Earth” and, in their figure (Zhao et al. 2022: fig. 3A), these latter authors showed soil taxa at > 10× that of all aquatic species. In other words, > 90% of biodiversity is present in Soil vs Ocean. Independently, around the same time, Blakemore (2022) estimated the “Soil Realm” is home to ~ 2.1 × 10^24^ taxa, or > 99.9% of global biodiversity, mostly Bacteria/Archaea or other microbes (excluding viruses), based upon published reports and extrapolation of topographic field data. Thus, rather than doubling it to ~ 50%, Anthony et al. (2023) actually halved soil biodiversity from > 99.9%. It is also remarkable that Decaëns et al.’s limited review claiming 23% biota seemed acceptable, unchallenged from 2006 because, instead of appraisal of realistic totals, it merely reported intensity of animal study, notably with terrestrial arthropods or aquatic species greatly overrepresented thus appearing disproportionately high. Such issues require critical re-evaluation and restatement of mainly microbial biota, as is attempted herein.

**Figure 2.**
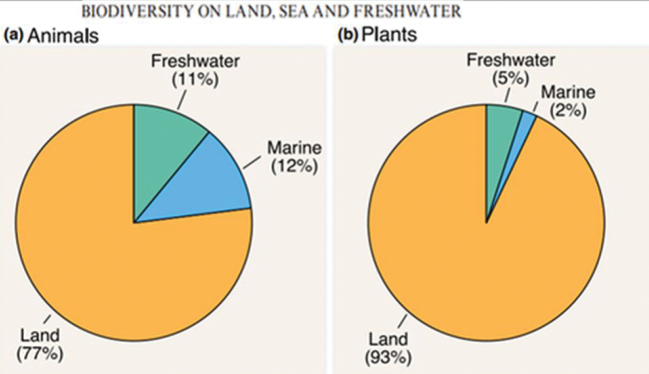
Román-Palacios et al. (2022: fig. 1) summed ~ 1.9 million extant animal and plant species combined, with ~ 80% on Land (~ 1.5 million species) without differentiating those found in Soil. Prokaryotic microbes were not included in their datasets, massively diminishing their terrestrial components (cf. Fierer et al. 2007). Lower still than in Freshwater, speculative claims that the Ocean supports > 80–99% of global biodiversity are readily dismissed by such solidly grounded facts.

In 1994 Robert May had assessed ~ 85% of all species as terrestrial (May 1994), and Benton (2001: table 1) extrapolated life on the Land to 12 million species, then being as much as 25× as diverse as in the Sea (just 0.5 million species), i.e., > 96% species on the Land vs < 4% in the Sea. Grosberg et al. (2012: table 1) found most macroscopic organisms were land-based (80%) compared with few in the oceans (15%), and fewer still in freshwater (5%). A recent status paper, like Decaëns et al.’s by Román-Palacios et al. (2022) had only ~ 2 million known species with 80% animals vs 20% plants, plus microbes and fungi needing to be added(!). Claiming combined relative proportions on the Land vs Aquatic of 78% vs 22% (Fig. 2), these authors yet failed to differentiate, making no mention of the Soil nor extrapolating likely totals, again downplaying soil biotic scope.

**Figure 3.**
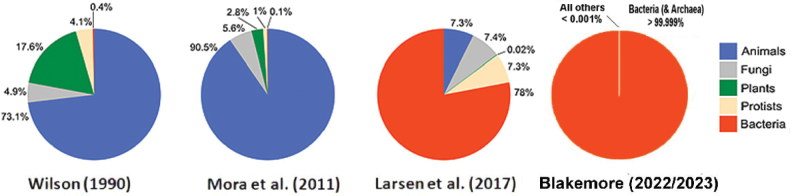
“Micro monde” progressions with microbial proportions greatly increased from Blakemore (2022, 2023: table 1, fig. 9) after Larsen et al. (2017: fig. 1). Of note, Larsen et al. (2017: tabs 1 and 4) in Scenarios already had Bacteria with ≤ 91% of total at up to 5.2 × 10^9^ taxa, compared to Anthony et al.’s (2023) 4.3 or 10 × 10^8^, these being mainly terrestrial, parasitic, or pathogens related to soil animals.

Further refinement of these Land vs Aquatic proportions was determined by Blakemore (2022) stating: “Based on topographic field data, an argument is advanced that Soil houses ~ 2.1 × 10^24^ taxa and supports > 99.9% of global species biodiversity, mostly Bacteria or other microbes. Contradictory claims that Soil is home to only a quarter of biota while Ocean harbors 80–99% of Life on Earth are both dismissed.” This statement requires clarification against Anthony et al.’s (2023) assertion that Soil hosts around 59% of species whereas their tables show only 10–50% (as tabulated below). Halving true proportions, their data totals are underestimated by orders of magnitude, seemingly due to them using older microbial count sources that have now been far superseded (Fig. 3).

More than a decade ago, prior to Larsen et al. (2017), a call for a “Census of Soil Invertebrates” (CoSI) catalogued 210,000 known soil species (Blakemore 2012: table 1) itself downplaying most microbes. An updated version had > 315,000 soil organisms (Blakemore 2016), but this also tallied just a sixth of total taxa as then known, albeit with massive proportional unknowns (Table 1).

**Table 1.** A 2016 “Census of Soil Invertebrates” (CoSI) with counts, mass, and diversity of common soil species.

Soil invertebrate group	Counts (mean) m^-2^	Biomass (range) g m^-2^	Total known species	% known
Viruses*	?	?	≈ 2,000–4,577	< 0.5%?
Bacteria and Archaea*	10^12^	20–500	≈ 7,500	< < 1%?
Fungi*	(500+ several km hyphae)	20–500	≈ 80,000	0.5%
Protozoa*	10^10^	6–30	1,500	8%
Rotifera (Bdelloid soil rotifers)	10^5^	?	300	?
Nematoda	10^6^	1–30	25,000	“*1.3*%”
**Lobopodia**			~ **1,200**	**< < 50**%
Lobopodia (Onychophora)	?	?	< 200	50%
Lobopodia (Tardigrada)			~ 1,045	?
Arachnida, Opiliones			6,300	?
Arachnida, Pseudoscorpionida			3,300	?
Acari (mites)	10^4^	0.2-4	45,200	4%
**Hexapoda (totals)**	**10^4^**	**0.2–4**	~ **9,000**	**17**%
Hexapoda (Collembola)	≤100,000		6,500	
Hexapoda (Diplura)			800	
Hexapoda (Protrura coneheads)			731	
Soil Insecta and their larvae	50–500	4.5	55,000+?	20%?
**Myriapoda (centi-, milli-pedes)**	**100–1,100**	**1.5–22.5**	**18,000**	**20**%
Myriapoda (Symphyla)			*200*	
Pauropoda (Myriapoda relative)			*700*	
Isopoda (slaters, woodlice, etc.)	≤ 1,800	< 4	5,000	?
Isoptera (termites)	Colonies	?	2,600	60%?
Blattodea (cockroaches)	?	?	4,500	?
Ants (Hymenoptera/ Formicidae)	Colonies	?	13,000	50%
Molluscs (soil gastropods)	?	?	24,000	40%?
Land Turbellaria (planarians)	?	?	830+	?
Terrestrial Polychaeta	?	?	?	?
Oligochaeta (megadriles + mostly aquatic microdriles)**	**50**–**5,000**	**20**–**500**	**10,000**	**20**%?
Microdriles (Enchytraeidae)***	1,000–300,000	1-53	~ 700	?
Microdriles (non- enchytraeids) in sodden, waterlogged, or wettish soil	?	?	1,000–2,300?	?
Megadriles (“true” earthworms)	50–4,875	20–500	~ 7,000	< 20%?
**Total species (approximate)**			**315,500**	**< < 1**%?

**Figure 4.**
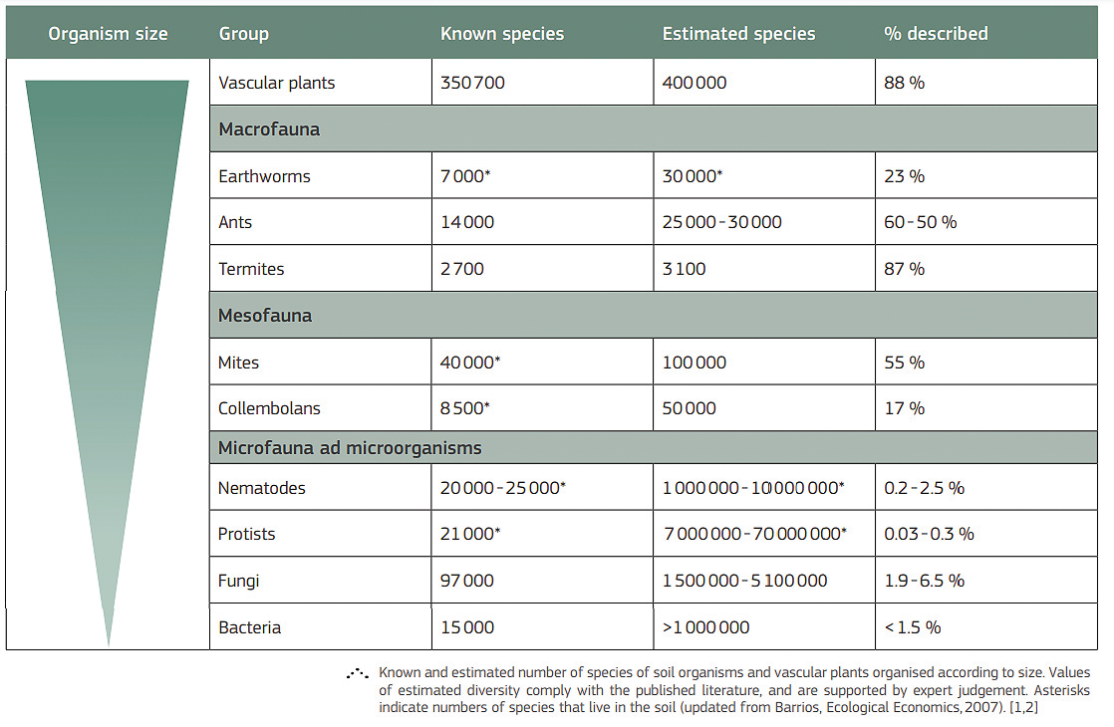
Global Soil Biodiversity Atlas (GBIF 2016) reporting ~ 667,000 soil biota or just about one third of known 2 million (much above Decaëns et al.’s (2006) 25% total!). Note that earthworms have 7,000 known and > 30,000 estimated species. Bacteria had 15,000 known species but estimated over one million (< 1.5% described). However, when microbes (excluding viruses) are properly considered and counted, as herein, soil unknowns are much higher (likely just < 0.0001% known at best). Vascular plants add ~ 400,000 species (cf. Anthony et al. 2023 with 466,000 angiosperm “Plantae”).

Table after Blakemore (2012: table 1, 2016: table 3) “from Brusaard et al. 1997; Wall and Moore 1999; Chapman 2009; Turbe et al. 2010: table 1; Fierer et al. 2007; Blakemore 2012; Wiki (https://en.wikipedia.org/wiki/Global_biodiversity#cite_note-col2016-5) and Pers. Obs.”. Fungal hyphae are from https://www.fao.org/agriculture/crops/thematic-sitemap/theme/spi/soil-biodiversity/soil-organisms/by-type/fungi/en/. *These taxa are especially revised upwards in the current study. ** Earthworms from Gobat et al. (2004: table 2.11, p. 42) of ≤ 500 g m^-2^ (to depth?) wet wt. so approximately half for dry weight and a quarter of this for Carbon: ~ 125 g m^-2^ C. Lee (1985: table 7) highest earthworms in NZ pastures (2,020 m^-2^, 305 fresh g m^-2^ “from McColl and Lautour 1978”); Coupland and McDonald (2008) report *Pontodriluslitoralis* (Grube, 1855) at 750–4,875 m^–2^. An Earthworm ratio of < 20% known gives expected total (7,000 × 5 =) ~ 35,000 spp (as also in Fig. 4). *** Enchytraeid maxima from Cragg (1963: table 2), Springett (1967: fig. 24) at Moor House, UK; Lavelle and Spain (2001: 281) also reported ≤ 93,600 m^-2^ at Point Barrow, Alaska.

Regarding Table 1 data, it may be noted that higher organisms host many unique symbionts or parasites and, as for microbes, many specific viruses too. For earthworms, Lee (1985: table 7) had highest numbers in NZ pastures (2,020 m^-2^ with 305 g m^-2^), higher counts are for littoral *Pontodrilus* sp. in WA. Note too that a total number of megadrile earthworms was predicted at ~ 35,000 species. Earthworms are important, as is noted later, due to their activities that greatly enhance other soil biota/microbes. Regarded as superficial soil-dwellers, “Soil Gastropods” were tabulated. However, viruses were only provisionally included as they fail to meet all criteria of independently living organisms, albeit they are included in several more recent biodiversity surveys. If such a line of argument for viruses were followed, then may not eukaryotic endosymbionts that only actively exist within host cells (Sagan 1967), with their unique genomes, be similarly added in biodiversity totals?

Contemporaneous to CoSI, a Global Soil Biodiversity Atlas (GBIF 2016: table 1) tallied 219,000 soil fauna/microbes while adding 350,700 vascular plants – on a premise plant seeds and roots are grounded in soil – raising totals to 667,000 soil taxa or roughly a third of all ~ 2 million species formally described as that time (Fig. 4).

At around the same time, a 10-year, $1 billion, Census of Marine Life (CoML 2010) funded 2,700 researchers at 670 institutions from > 80 nations to conclude a total of just ~ 230,000 Ocean taxa (or ~ 12% of the 2 million known species!). They claimed this was just one tenth of Ocean’s expected total of another two million species, hence a new Ocean Census project “launched” on 27 April 2023 to net the remainder. A similar 2011 Census of Deep Life (CoDL), a central pillar of the Deep Carbon Observatory (DCO - https://deepcarbon.science/), investigated diversity, distribution, and biogeography of obscure subsurface biospheres having little relevance to evolution or extinctions.

As argued in the current report, such expensive sub-marine projects distract funds and efforts from surveys of more crucial soil biota that are much less well-known and more endangered, extinctions being time critical. How is it justified to fund long-term abyssal taxonomy at $ millions per species while unknown soil taxa, that may be easily sampled in the field with a spade, are being extincted?

Although primarily concerned with rapid advances in molecular analyses (“Omics”) revealing microbial diversity increased by several orders of magnitude (as detailed herein), lesser concerns are upping of counts for topographical terrain and delving into soils to full depth. However, unlike routine biotic surveys via planimetrically flat transect, plot, or quadrat, some surface-area independent inventories (e.g., of farm stocks or people) do not gain from realistic terrain extrapolation, neither do level waterlogged entities (e.g., lakes, mires, or bogs).

In general, prior to 2018 almost all soil inventories were based upon unrealistic, planimetrically flat land areas, thus true soil counts are likely more than doubled, and possibly quadrupled, when properly allowing for terrain and microtopography overlays (Blakemore 2018b), reducing further the marine majority claims. Although such work shows Soil is clearly more crucial and diverse, due to lack of equable support or funding, less than 1% of its meso- and macro-faunal organisms are as yet unearthed (FAO 2020). Furthermore, only a tiny fraction of the enormous soil microbiome is identified, with the proportion of known soil microbiota likely much < 0.0001% (as per Blakemore 2023), thus most of the vast array of Soil Biota remain an unexplored mystery awaiting discovery.

**Figure 5.**
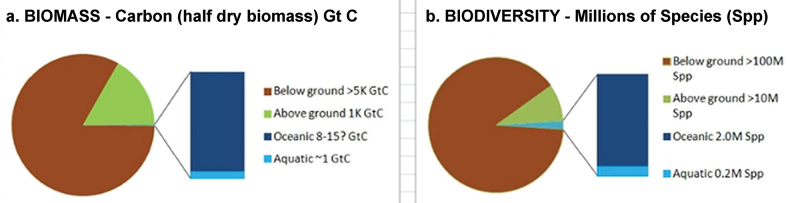
Global biomass (plus dormant/ necromass) and biodiversity in context of biome proportions (from Blakemore 2023 after https://vermecology.wordpress.com/2020/05/27/realms-of-the-soil/: fig. 2 and https://veop.files.wordpress.com/2022/09/new-addendum-file.pdf: fig. 4), being updated in the current report. In the figures above “below ground” or sub-surface refer to soil biotic activity related to surface productivity and not to the deeper subsurface biota.

Moreover, rather just scratching the surface to cm or a metre deep, recent studies have mean depth-to-bedrock at 13.1 m plus friable saprock may add 8 m to total > 21 m soil depth (Shangguan et al. 2017: table 1; Hicks-Pries et al. 2023; Blakemore 2024). Consequently, most soil estimates, including those herein, may be an order of magnitude too low and hence the relative soil biomass and diversity values as in Fig. 5 (cf. Fig. 2) are most modest. This dependent upon estimates of full soil depth.

Extrapolation of soil sampled at just a few superficial centimetres or a metre, to allow for full depth (≤ 21 m as noted above) are not yet applied but in themselves may increase soil stocks by an order of magnitude. Rolando et al. (2021) found soil layers below 90 cm up to 5 m deep accounted for 80% biomass, while the 0–30 cm layer represented only 10% of total soil carbon (i.e., × 10 for > 30 cm).

A further distinction is definition of “deep subsurface” biota that source energy differently to subsoil species. Beaver and Neufeld (2024) state that there is no universal depth that defines the terrestrial subsurface biome, previous publications having described “terrestrial subsurface as deeper than 8 m, and the deep terrestrial subsurface as deeper than 100 m.” For the purposes of their review, the deep terrestrial subsurface comprised of rocks and groundwater at least 100 m below the surface of the Continents. Bar-On et al. (2018) “define deep subsurface as the marine subseafloor sediment and the oceanic crust, as well as the terrestrial substratum deeper than 8 m, excluding soil.” An important demarcation, although unapplied in the present review, is soil biotic biomass and biodiversity to whole mean depth of soil activity (or frozen in Permafrost), now globally averaged near 21 m.

Soares et al. (2023) suggested 12–20 % of Earth’s biomass exists in the terrestrial deep subsurface, compared to ~ 1.8 % in the deep subseafloor, further confirming “terrestrial deep subsurface holds ca 5-fold more bacterial and archaeal biomass [thus, by proxy, biodiversity?] than the deep marine subsurface.” Although the total Ocean biota is again diminished, this deep subsurface data is a much lesser concern in the current global review of Soil biota and is only briefly mentioned in passing.

Abundance of biota relates to both its biomass (living, dead, or dormant forms) and its biodiversity species counts. Initially, a preliminary global microbial abundance estimated by Whitman et al. (1998: tabs 3–5) was 2.6 × 10^29^ vs 1.2 × 10^29^ cells in Soils vs Aquatic (marine and freshwater) habitats, and 26.0 vs 2.2 Gt C biomass, respectively. This was an indicator that Soil clearly supports twice the Ocean biota, and ten times its biomass as an early realization that Soil likely supports > 50–90% of Life on Earth. Deep sub-surface microbiota, which are largely irrelevant to most active above-ground Earth processes, were 3.6 × 10^30^ vs 2.5 × 10^30^ cells in Oceanic vs Terrestrial sub-surfaces. However, revisions by Kallmeyer et al. (2012), Parkes et al. (2014), Magnabosco et al. (2018), and Hoshino et al. (2020) had just 3–5 × 10^29^ vs 2–6 × 10^29^ cells (biomass of ~ 4 vs 23–31 Gt C), respectively. A global tally of ~ 10^30^ cells was determined independently by Blakemore (2022, 2023), but for somewhat different proportions, for reasons as explained and briefly restated herein.

Soil was shown with ≤ 10^8^ –10^12^ cells/g dry weight or 10^14^ –10^18^ cells/t, there being 10^6^ grams in a tonne. Biodiversity ranges were 10^2^ –10^6^ species/g or 10^8^ –10^12^ species/tonne of soil. Global topsoil was calculated as ~ 2.1 × 10^14^ t to 1 m depth. Therefore, the total ranges were 2.1 × 10^28^ –10^32^ cells (median ~ 2.1 × 10^30^) and 2.1 × 10^22^ –10^26^ soil species (median ~ 2.1 × 10^24^). Having a new mean soil depth of ~ 21 m would possibly increase these by an order of magnitude, but is not yet applied. Comparatively, Anthony et al.’s (2023) global species total (10^10^) is mid-range in the biodiversity of a single tonne of topsoil. Moreover, an equivalent to all the Oceans’ biodiversity may similarly be held in just a handful of fertile topsoil, or much less than a tonne, albeit, as a general “rule of thumb”, a dry tonne of topsoil occupies 0.65 cubic metres, a ground area of < 1 m^2^, or a small step for a man.

Prior sources had determined: “species of bacteria per gram of soil vary between 2,000 and 8.3 million” (Gans et al. 2005; Roesch et al. 2007) (= 10^4^–10^6^ spp/g or 10^10^–10^12^ spp/t that, if all unique taxa, is equivalent to twenty billion or up to a trillion species per topsoil tonne). Discrepancies in Gans et al. (2005) are samples of 10 g soil so strictly 0.83 × 10^6^ spp/g, yet their fig. 4 shows total species number computed as ≤ 10^7^ thus a million or so spp per g seems correct. Raynaud and Nunan (2014) had: “a single gram of soil can harbour ≤ 10^10^ bacterial cells and an estimated species diversity of between 4 × 10^3^ to 5 × 10^4^ species” (= 10^14^–10^16^ cells/t and 4 × 10^9^–5 × 10^10^ spp/t). Bickel and Or (2020) found: “bacterial phylotypes ranges between 10^2^ and 10^6^ per gram of soil, with high values similar to the diversity in all of earths environments” (= 10^8^–10^12^ spp/t). James et al. (2022) summarized: “Soil microorganisms are the largest biodiversity pool on earth, with more than 10^30^ microbial cells [total surely!?], 10^4^ –10^6^ species, and nearly 1,000 Gbp of microbial genome per gram of soil”. Although fully extrapolated values from the cited reference sources are listed, only the median of the various value ranges are taken as reasonable summaries, these being presented herein.

As already noted, using scaling values, Zhao et al. (2022) found: “Although the estimated total abundance of global airborne bacteria (1.72 × 10^24^ cells) was 1 to 3 orders of magnitude lower than that of other habitats, such as soil (9.36 × 10^28^ cells), freshwater (4.70 × 10^25^ cells), and marine (4.68 × 10^28^ cells) habitats, estimates of the bacterial richness of the atmosphere (4.71 × 10^8^ to 3.08 × 10^9^) were comparable to those of the hydrosphere”. In other words, they confirm Soil at ~ 10^29^ with twice as many microbial cells as the Ocean and, whereas their figure (Zhao et al. 2022: fig. 3A) shows a richness of > 10^11^ soil microbe OTUs, the Ocean or Freshwater and the Air each only have ~ 10^10^ taxa (< 10%). This translates as Soil housing ~ 90% of global biodiversity, as indeed May (1994) had intimated 30 years ago, before the scope of microbial megadiversity was realized as being so vast.

For microbial diversity, recent developments of rapid genomic sequencing and bioinformatics (-omics) allow scaling values such as by Locey and Lennon (2016) to show Earth with ~ 10^12^ microbial OTU taxa (just 10^10^ or ~ 1% in global Ocean). These totals were soon raised to 10^12^– 10^14^ microbial taxa by Lennon and Locey (2020) and then by Fishman and Lennon (2022) who had “a soft upper constraint of 10^22^–10^23^ due to neutral drift” for all taxa. Their upper boundary is increased by 20× for median species total in the current study and, regardless of scaling values, confirm Soil’s > 99.9% of global biodiversity, being almost entirely microbial. These authors’ soft upper constraint of 10^22–23^ taxa dispersed in 10^29–30^ soil cells is a ratio of one taxon per ~ 10^6–8^ cells.

**Figure 6.**
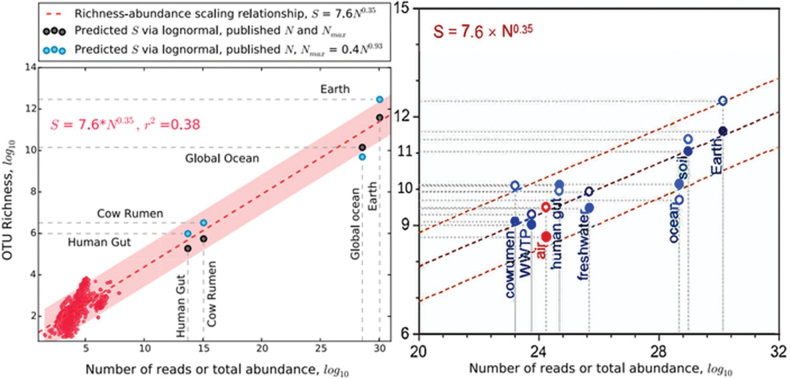
Relative microbial abundance vs diversity after Locey and Lennon (2016: fig. 3), and Zhao et al. (2022: fig. 3A) who added wastewater, air, freshwater, and soil. The Ocean has < 1% of global biodiversity, barely above freshwater or air, and less than the human gut biome! The soil microbiome is revised upwards in Table 2 as its abundance vs richness apogee peak is more extensive than any other major (or minor) habitat.

Summarizing the microbial status, Zhao et al. (2022) said: “soil is the most microbiologically abundant (10^29^) and diverse (10^11^) environment on the Earth”. Although their cell count may be within bounds, their diversity – albeit ~ 10× greater than the Ocean’s – is disproportionately low due to incomparability of Soil’s scaling ratio when compared to any of their other habitats (Fig. 6; Table 2) .

**Table 2.** Prokaryote proportional counts and biomass in Earth’s six major ecological Realms-of-Life.

Ecological realm	Cells/CFUs × 10^28^ (%) *	Species/OTUs (%) *	Biomass Gt C (%)
1 Soil *	210 (56%)	2.1 × 10^24^ (99.99%)	~ 209.6 (56%)
2 Land superficial **	100 (27%)	10^12^ (< 0.001%)	~ 100? (27%?)
3a Land subsurface ***	~ 20–60 (11%)	< 10^5^	~ 23–31 (7%)
3b Marine subsurface ***	~ 2.9–35 (4%)	< 10^6^	< 35 (9.3%)
4 Ocean **	12 (3%)	10^10^ (< 0.0001%)	0.6–2.2 (0.5%)
5 Aquatic on Land **	< 0.02 (< 0.005%)	< 10^10^ (< 0.0001%)	0.3? (< 0.1%?)
6 Atmosphere ****	(10^24^)	(10^8^–10^10^)	? (< 0.0001%?)
TOTAL	~ 378 × 10^28^ (100%)	~ 2.1 × 10^24^ (100%)	~ 373 (100%)?

* Data from Blakemore (2022, 2023: table 2) greatly modified from Whitman et al. (1998: table 5). Fishman and Lennon (2022) had: “bacterial and archaeal taxa S_present_ is between 10^6^ and 10^23^”; at ~ 2.1 × 10^24^ soil taxa their upper value is increased by twenty times (Blakemore 2022). CFUs = Colony Forming Units (microbial), OTUs = Operational Taxonomic Units (genetic). Soil microbial biomass is updated to 209.6 in Appendix 1. ** Data extrapolated from Zhao et al. (2022: fig. 3A), Locey and Lennon (2016: fig. 3), Lennon and Locey (2020), and Whitman et al. (1998) who had aquatic habitats, mainly Ocean, with 0.6–2.2. Gt C (just 0.15–0.55%). Grosberg et al. (2012) estimated aquatic habitats occupying ~ 1–2% of land area (now halved to 0.8% due to terrain!) have one-third of the Ocean’s biodiversity (and hence likely one-third of its biomass?). *** Revision of subsurface by Kallmeyer et al. (2012), McMahon and Parnell (2013: table 3), Magnabosco et al. (2018: fig. S23), Bar-On et al. (2018), Hoshino et al. (2020), Blakemore (2022, 2023), Soares et al. (2023). **** Total 10^6^ cells/m^3^ to 1 km altitude (https://en.wikipedia.org/wiki/Aeroplankton) gives 10^24^ cells (> 10^10^ spp?); however, microbes, including Bacteria and Fungi, have been detected in the atmosphere at high altitudes making the atmosphere the Earth’s largest biome – much greater than was claimed for the Ocean. Contrary to such Ocean claims, Whitman et al. (1998) said: “By volume, the atmosphere represents the largest compartment of the biosphere, and prokaryotes have been detected at altitudes as high as 57–77 km”. Zhao et al. (2022) support these earlier contentions: “While the total abundance of global airborne bacteria in the troposphere (1.72 × 10^24^ cells) is 1 to 3 orders of magnitude lower than that of other habitats, the number of bacterial taxa (i.e., richness) in the atmosphere (4.71 × 10^8^ to 3.08 × 10^9^) is comparable to that in the hydrosphere”. Naturally, many Aeroplankton taxa are shared with the Phytoplankton and Phytomenon. Zhao et al. (2022: fig. 3A) (Fig. 6) also show a human gut biome has greater biodiversity than all the hydrosphere (the realization of which many marine or freshwater researchers may find particularly difficult to stomach). As already noted, aquatic or deep sub-surface biota are of less practical concern to the current study on Land and Soil organisms, although they again highlight deficiency of Ocean’s excessively claimed biota at all scales and at all depths, almost all being downgraded in subsequent reviews supporting the need for a “sea change” of appreciation and much increased support for soils.

Table 2 contextualizes a current estimate of 2.1 × 10^24^ soil taxa as 99.99% of a global total. Species values presented herein (e.g., Fig. 6, Table 2) may be contrasted to microbial Spp/OTU counts in Anthony et al. (2023: table 1), arranged in a slightly revised format for better clarity of comparison, as shown in Table 3.

**Table 3.** Species (Spp/OTU) biodiversity key values re-formatted from Anthony et al. (2023: table 1).

Biodiversity spp/OTUs *	Lower × 10^8^	Central × 10^8^	Upper × 10^8^
**EARTH**			
“Phage”	1.000	1,000.0	3,700
Microbe total **	0.067	10.1	10,000–1,000,000
(Microbe just Bacteria)	(0.044)	(10.0)	(37)
**Earth total**	**1.10**	**1,010.1**	**3,740** ***
Earth non-Phage total	0.100	10.1	40
Earth non-Phage, non-Bacteria	ND	0.1	ND
**SOIL**			
“Phage”	0.056	99.0	1,590
Microbe total **	0.060	4.4 ****	“?”
(Microbe just Bacteria)	(0.010)	(4.3)	(33)
**Soil total**	**0.095**	**104.0**	**1,620**
Soil non-Phage	0.039	5.0	30
Soil non-Phage, non-Bacteria	ND	0.7	ND
% **Soil vs Earth totals**			
Totals	8.0%	**10.3**%	43.3%
Totals non-Phage Totals non-Phage, non-Bacteria	39.0% ND	**50.0%** [-86%!]	75.0% ND

*10^8^ is 100,000,000 species (Spp) or operational taxonomic units (OTUs). ** Microbe totals are for “bacteria, archaea, and fung*i*”, but the non-Bacteria values are seemingly erroneous as Soil (0.7) has more than all Earth (0.1). *** Upper value “3.74 × 10^11^” ignores Microbes with “10^12-14^” taxa. **** Cf. Zhao et al. (2022) have > 10^11^ for soil and > 10^12^ for Earth, and Blakemore (2022, 2023) has total microbes 2.1 × 10^24^ (cf. Table 2) plus total global viral/phage count (as presented herein) of ≤ 10^26^ taxa, found mainly in Soil (see text for details).

Deep carbon data are of less practical concern to the current study on Land and Soil carbon stocks and cycles, although they again highlight deficiency of Ocean’s excessively claimed biota at all scales and at all depths, almost all being downgraded in subsequent reviews.

**Figure 7.**
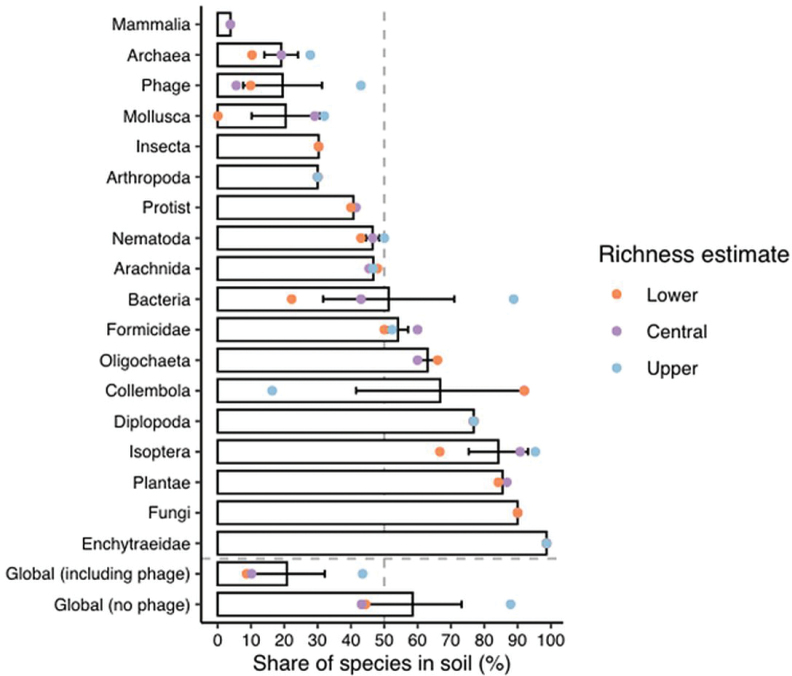
Unsystematically selected taxonomic groups in Anthony et al.’s (2023: fig. 2) (cf. Table 1) appear to show Bacteria’s Upper dominance at > 90% in both Soil and “Global (no phage)” totals but, strangely, they omit Megadrile earthworms being > 99% resident in soils, as their name would suggest and as restated below. Comparatively, their Enchytraeidae, according to Martin et al. (2008), are < 47% terrestrial, not 99%!

Anthony et al. (2023: fig. 2) confirm Bacteria richness ≤ 90%, proving their dominance in Soil (Fig. 7) but are mistaken for Oligochaeta, as Earthworms are truly higher with 99% soil occupancy which as is discussed further in the review section below.

## ﻿Materials and methods

The intention of this review is to compile and compare recent Soil Biota studies by Blakemore (2022, 2023) with Anthony et al. (2023). In the last few decades, the advent of high-throughput DNA amplicon sequencing and rapid genetic analyses (-omics) has revealed the complete dominance of microbes in biotic tallies, especially in soils, and a need for realistic biodiversity estimation from projections of their unknown and undescribed components. Realizing our ignorance of soil microbes exposes a stark disparity: Most accounts of global richness reflect historic intensity of study rather than relativistic estimates due to irrational fact of overwhelmingly research effort and funding directed into Aquatic, Oceanic or Space research (e.g., NASA, JAMSTEC, NOAA, Scripps, Woods Hole, https://en.wikipedia.org/wiki/List_of_oceanographic_institutions_and_programs), not Soil. The International Union of Soil Sciences (https://www.iuss.org/) does not list any dedicated institute.

The summary of progress in relative soil biodiversity studies, as introduced above, is further reviewed and where necessary corrected, mostly for microbe counts but also to allow for terrain (after Blakemore 2018b). Factoring soil depth may further double numeric values if not exponentials.

In addition, several omissions and uncertainties (“?”) from various published sources are clarified.

## ﻿Body of review

### ﻿Regarding Anthony et al. (2023) soil enumeration values questioned with “?”

For Mammalia, Anthony et al. (2023: table 1) had Lower to Median ranges with 75–250 soil species, yet their Upper range was marked “?”. Although relatively unimportant, a nominal value in Decaëns et al. (2006: fig. 2) of ≤ 1,000 soil mammals may be a reasonable estimate for this well-known fauna.

However, Anthony et al. (2023) define soil species as “those that live within, on (e.g., insects that feed on the surface of soil), or which complete any part of their life cycle in soil (e.g., organisms with an inactive pupal stage in soil or plant seeds that germinate in soil) or in the tissues of soil-dwelling symbionts (e.g., microbial parasites of soil animals).” Hence, it may be moot to extend inclusion to almost all terrestrial mammals (except, perhaps, some wholly arboreal or semi-aquatic species) that live or feed on soil (or end their life cycle often buried, inhumed, or interred therein!), e.g., *Homo*.

Secondarily, encompassing of many (most?) insects within the definition of soil species adds to an argument for the inclusion of Larsen et al. (2017) insect parasites or pathogens as part of Soil Biota.

Other groups in need of more pertinent “?” clarification are presented in sequential order below.

### ﻿Regarding Annelida: Oligochaeta (true earthworms and their lesser relatives)

Anthony et al. (2023: table 1) have “?” questioning a possible Upper range of their Oligochaeta which is surprising since they cite the “Global Soil Biodiversity Atlas” (GBIF 2016 - https://esdac.jrc.ec.europa.eu/public_path/shared_folder/Atlases/JRC_global_soilbio_atlas_low_res-2019-06-13.pdf) that states: “Earthworms belong to the phylum Annelida (class Clitellata, subclass Oligochaeta). The Oligochaeta contain 10 400–11 200 species in approximately 800 genera, and 38 families comprised of approximately 7 000 true earthworms.” They seem to have missed the subsequent statement: “Although 7 000 ‘true’ earthworms (in 20 families) have been described to date, the total is probably around 30 000 species globally”.

This is clearly shown in the GBIF (2016: table data) that is reproduced here in Fig. 4.

Phylum Annelida includes Classes Oligochaeta (earthworms), Polychaeta (marine worms) and, erstwhile, Hirudinea (sanguinivorous or predatory leeches). Due to an inordinate amount of funding for marine research, ~ 13,000 polychaeta are now reported, but only ~ 8,000 are considered valid taxa; similar synonym statistics apply to earthworms but, due to their high endemicity and Soil’s heterogeneity, their unknowns are legion. The Oligochaeta comprises mainly soil dwelling Order Megadrilacea from Benham (1890) – the “true” earthworms – and his Microdrilacea for smaller, mainly aquatic worms. Strangely, in Anthony et al. (2023: table 1) their “Oligochaeta” has between 5,000–10,000 total taxa (apparently sourced from Martin et al. 2008 and a GBIF Checklist) and they further claim 3,300–6,000 Oligochaeta in soil (from Martin et al. 2008 and Decaëns et al. 2006). Contrary to Anthony et al. (2023: fig. 2) (see Fig. 7), Martin et al. clearly stated: “Most microdriles are fully aquatic, with the exception of the Enchytraeidae, a family that is primarily terrestrial; of the 650 described species, 200 are aquatic and 150 marine”, or primarily > 52% aquatic! These relative figures are treated in further detail below as it is important for facts to be both current and correct.

Martin et al. (2008: table 1) did indeed claim only 5,000 valid species of Oligochaeta*s. stricto* and said 4 of the 14 megadrile families (in actuality six of twenty families) have aquatic or semi-aquatic species (or, for *Pontodrilus* spp., littoral). They further state that “No fewer than 60 species of megadriles are also considered aquatic” and list total aquatics {in squiggly braces} in these stated genera as: Almidae {41 spp.}, Criodrilidae {2}, Lutodrilidae {1}, Sparganophilidae {14}, plus several Lumbricidae claimed to be frequently found in aquatic situations (although this may be questioned as it is often adventitious rather than fixed). Surprisingly they omit other megadrile genera with aquatic species such as Megascolecidae (e.g., a few in NZ lakes) and Pontodrilidae {2 spp.} that is wholly littoral. This biodiversity data requires updating since at least 7,000 truly megadrile taxa are currently described (see Blakemore 2000, 2008, 2016), and whereas names are continually added the more we search and discover, probably less than 20–30% of all species are known, as found by Lee (1959) in New Zealand (cf. Glasby et al. 2009; Blakemore 2011), and by Blakemore (2000) in Tasmania. Numbers of synonyms are un-estimated while likely cryptic species need clarification. If their relative proportions hold true, as Blakemore (2022) suggested, then the average of six cryptics per morphologically described arthropod taxon as in Larsen et al. (2017: table S1) quite counterbalance the ~ 18% eukaryote synonyms that were estimated by Mora et al. (2011).

Anthony et al.’s (2023) preliminary research also overlooked Australian ABRS (2009) global summary with: “7,684 Oligochaeta from Blakemore (2008 and pers. comm.)” and around 30,000 total anticipated global species. As Blakemore (2013) explained, hierarchical classification of true earthworms is: Annelida Lamarck, 1802; Oligochaeta Grube, 1850; Megadrilacea Benham, 1890 with ~ 20 or so families including Moniligastridae, Ocnerodrilidae, Acanthodrilidae, Exxidae, Octochaetidae, Megascolecidae, Lumbricidae and Eudrilidae (all *sensu*Blakemore 2000). Thus, Megadriles have ~ 7,000 known species (with cryptics cancelling synonyms?) compared to mainly aquatic Microdrilacea, composing around 2,300 spp. (Table 1) plus a quite minor microdrile family that these authors – for some unsystematic reason – gave great import: Viz. Enchytraeidae with only around 700 species. Whereas Anthony et al. (2023) claim this family is the most wholly soil-dwelling group with “98.6%” terrestrial members, this is misconstrued as the majority of this Microdrile family is fully- or semi-aquatic; being small, pale and relatively ineffective. Microdrile researchers are classed as aquatic workers, rather than true soil-based, Megadrile eco-taxonomists, consequently they too appear to enjoy greater support and funding for seemingly obscure reasons.

A summary of relative abundance and biodiversity of these Oligochaeta is compiled in Appendix 1.

Another source is García-Roselló et al. (2023: table 1) GBIF database of Annelida: Clitellata with only 8,000 total species but which falsely claims 13.6% are Marine. In comparison, Anthony et al. (2023) strangely state: “Annelids, including the Enchytraeidae and Oligochaeta, with the lowest overall biodiversity but high specializations to soil. We estimate that there are 7.8 × 10^2^ and 1 × 10^3^Enchytraeidae and Oligochaeta species and that 98.6 ± 0.06% and 63 ± 4.2% of species live in soil, respectively.” We may graciously accept this in part as a typing error since the most basic of research reports frequently cite over ~ 7,000 described Megadrile Oligochaeta alone, not just 1,000. Moreover, rather than just 63%, a majority of Megadrile Oligochaeta being wholly soil dwellers is closer to > 99%, as the name, ‘Earthworm’, suggests (cf. Fig. 7). Although obvious, this is restated.

Thus – contrary to Anthony et al.’s (2023) indication – most of the true earthworm families are terrestrial and nearly 100% resident in soils. Martin et al. (2008) citation of 60 wholly aquatic megadriles may be a reasonable number, that – in a megadrile group of ~ 7,000 taxa – is < 1% making them one of the most specialized of committed soil residents. Other candidates such as the termites or ants are insects living in colonies with winged stages (almost liken to soil “tourists”), thus not as highly endemic nor as specialized as earthworms are. Other taxa such as hexapod Collembola or Acarid mites are typically superficial soil/litter dwellers and depend upon earthworm burrows for their soil ingress. There are several others of less populous soil faunal and floral groups that may also have 100% edaphological species. For instance, components of the ubiquitous superficial cryptogamic Biocrust or extensive Phytomenon that, as well as being most ancient flora, may rival marine Phytoplankton for abundance, diversity, as well as for NPP productivity (Blakemore 2024).

Phytomenon is a recent term for microscopic “plants” that abide, as is appropriate for terrestrial single-celled autotrophs, compared with the marine or aquatic Phytoplankton (“plants” that drift) or the Aeroplankton (aerial floating microbes) as noted already (see Blakemore 2019, 2023, 2024).

### ﻿Regarding soil Bacteria (plus Archaea)

Anthony et al. (2023: table 1) had Bacteria included within their Microbes often marked with a “?”. Global biodiversity is now dominated by Bacteria within the Soil Realm, as Blakemore (2022) showed, with new totals of ~ 2.1 × 10^24^ taxa in ~ 2.1 × 10^30^ cells indicating that one species, or operational taxonomic unit (OTUs), exists for around each 10^6^ cells. In this review a justified argument is that a unique taxon per million cells is reasonably applicable. As there is no central registry – nor yet a dedicated Soil Ecology Institute – diversity data compiled from diverse sources are updated or corrected as necessary in periodic reviews, such as this present contribution.

In Norway, Torsvik et al. (1990) had found ~ 1.5 × 10^10^ bacteria cells per gramme of dry forest soil distributed among 4,000 clones with standard genome sizes; a mean number was ~ 4 × 10^6^ bacteria per clone per g of dry soil. This indicated soil bacterial populations comprise many genetically separate clones, with a mean of around ~ 3.75 clones per million cells. This local data suggests more than one species/OTU per million cells is a reasonable approximation.

Worldwide, Roesch et al. (2007) estimated mean microbial populations limited to ~ 1 billion cells per g of soil (10^9^ cells/g) comprising 10^3^–10^6^Bacteria/Archaea species, or at least one and up to as many as one thousand species per million cells(!). They also found 2,000–10,000 species per gramme of soil were underestimates. Therefore, an extrapolated mean may be closer to 10^5^ spp/g (per 10^9^ cells/g), suggesting an average nearer to 100 Bacteria/Archaea species per million cells.

As early as 2008, Fulthorpe et al. (2008) had determined that ≤ 87.9% of Bacteria were unique to the soil they were sampled in, and only 1.5% were common to all soils across a large transect of American continents. The same does not hold for the Ocean that is much more homogenous, with intermixing biota widely dispersed. This was clearly shown by Louca (2022: figs 1, 2) with soil habitats four or more orders of magnitude more diverse than marine (etc.) habitats over shorter distances. Dispersal was slowest for terrestrial sub-surfaces, indicating mostly soil environments acting as “isolated islands” of endemic microbial evolution. His “hot-spring” data is interesting as, contrary to claims for Marine origin, most current information point to these being the font of all Life, consistent with Darwin’s prescient “Warm little pond” theory of Origin (e.g., Damer 2016).

Whereas Larsen et al. (2017) proposed a new Pie of Life projected for > 1–6 billion (10^9–10^) species on Earth dominated by Bacteria (~ 70–90% of total) which they mainly considered just for insect hosts, Bahram et al. (2018) concluded Soil as Earth’s most diverse biome but failed to give figures. For estimates of around 3 × 10^29^ cells in soils, Flemming and Wuertz (2019), as for Bar-On et al. (2018), also give no species data. Subsequently, Louca et al. (2018, 2019) claimed only “2.2–4.3 million full-length OTUs worldwide” (3 × 10^6^) refuting predictions that billions or trillions of prokaryotic OTUs exist. Yet Wiens (2023) explained how Louca et al. (2019) had made entirely avoidable underestimation errors whilst also revising Larsen et al.’s (2017) projected 1–6 billion estimate downwards to a modest 0.183 to 4.2 billion (10^8–9^) species with 58–88% Bacteria, but again most of these in insect hosts rather than in the much more diverse and extensive Soil habitat.

Conversely, Raynaud and Nunan (2014) said: “The application of novel molecular techniques (such as high throughput sequencing) during the past two decades has uncovered a phenomenal bacterial diversity in soils.” They quoted “a single gram of soil can harbour up to 10^10^ bacterial cells and an estimated species diversity of between 4·x 10^3^ to 5·x 10^4^ species”. But they also noted “when bacterial density is 10^9^ cells g^−1^ or less. α = 1107.53 corresponds to a species richness of 15000 species for 10^9^ cells whereas α = 264.79 corresponds to a species richness of 4010 species for the same number of cells.” This higher diversity of 4–15 species per million cells is medial to a range estimated earlier of an average one to 100 bacterial species per million cells in Soil as noted above.

At a trans-European transect scale, Plassart et al. (2019) extracted 3 × 10^6^ 16S rRNA sequences from 71 × 1 g (dry?) soil samples, detecting a total of 34,190 OTUs ranging from 653 to 1,860 (mean: 1,307) OTUs/g. This ~ 10^3^ taxa/g is low to midrange of totals as given elsewhere, possibly due to the methods, soils, or the local climate. Their rarefaction curves of bacterial OTUs followed a logarithmic model without reaching a rarity plateau. Higher richness estimates of between 590 and 100,000 species per gram (10^2–5^ OTUs/g) for similar 16S rRNA PCR sequences were reported by Schloss and Handelsman (2006), their lower range from a remote, presumably wintery, Scottish soil.

In harsh Alpine biomes, Adamczyk et al. (2019) still extracted an average of 1.7 × 10^4^ OTUs per 250 mg sample (thus about 6.8 × 10^4^ OTUs per g?), just a quarter fungal, and they also determined that soil acidity and elevation were the most deleterious variables in these extreme habitats.

Regarding rarity of soil species, Bickel and Or (2020) had most bacterial species classified as rare (99.6%) and these made up ~ 42% of a global relative abundance, they concluded: “The complex structure of soil pores offers numerous refugia for hosting diverse bacterial species. This wide range of microhabitats is particularly important for maintaining the rare components of the soil microbiome”. From their global microbial biodiversity of ~ 10,000 OTUs per g dry soil, since Soil harbors ~ 10^10^ cells per g, and these are mostly Bacteria/Archaea, this supports a reasonable average of around one OTU/species per million cells in Soil. Q.E.D.

Recently, Jia et al. (2022) and Sun et al. (2023) confirmed in quite local samples what Fulthorpe et al. (2008) found for trans-continental soils, with rare or unique bacteria being 90–98% while only a minority of species were common. This supports high Soil biodiversity at sample to Continent scale.

Soils naturally include a root-zone Rhizosphere: “the most diverse microbiomes on Earth, containing up to 10^11^ microbial cells and ~ 30,000 bacterial species per gram of root. The rhizosphere microbiome exists through an interwoven tapestry of bacteria, viruses, archaea, protists, fungi, nematodes, and small arthropods interacting directly with plant roots and each other” (White et al. 2021). McNear (2013) found 10^10^ –10^12^ cells per gramme of rhizosphere, endorsing 10^11^ cells/g as a reasonable, but higher, median count in this rich soil microhabitat compared to the Soil environs.

Almost all the studies above are consistent with Blakemore (2022) determining a modest one species per million cells (viz. 2.1 × 10^24^ species in 2.1 × 10^30^ cells in Soil globally). However, as noted, underestimations may be one or more orders of magnitude, so all values are approximate. The wide uncertainty range of 10^22^ –10^26^ total species (median ~ 2.1 × 10^24^) within 10^28^ –10^32^ cells (median ~ 2.1 × 10^30^) shown in this report is commensurate with previous estimations; compared to Fishman and Lennon (2022) the increase is around 20-fold. This is compliant with Bar-On et al. (2018) who had a 10-fold margin of error in their microbial estimations and a 32-fold error factor for viruses.

Fungal rarity ratios, when simultaneously studied, appear comparable with those for Bacteria, albeit fungal biodiversity, also mainly in soil, is often less by varying factorials (e.g., Labouyrie et al. 2023).

More support for higher Soil Bacteria diversity, both relative and compared to in any other habitat, are indicated by local and global Virus to Bacteria (VtB) ratios which will now be discussed further.

### ﻿Scaling the Virome – Virus-Like Particles (VLPs) and Virus to Bacteria (VtB) ratios

A virion is an infectious virus particle, while a virus-like particle (VLP) is a non-infectious nanostructure that mimics a virion, but often these terms are used interchangeably. “Phage” is used informally for a bacteriophage that infects and replicates within Bacteria or Archaea, often a synecdochal term for all viruses, not strictly correct thus only quoted and not self-applied in this review. Virus to Microbe (VTM), Virus-Bacteria Ratio (VBR) or Virus to Bacteria Ratio are also interchangeable expressions; hereafter only the latter (VtB) is used.

Tabulated VtB ratios are presented in Appendix 2, revised for microbial counts in Blakemore (2022, 2023), to give a global total of ~ 5.1 × 10^31^VLPs with ~ 4.1 × 10^31^ (~ 80%) virions in soils (to partial depth). This updates the soil virus value, allowing for non-ice and non-desert terrain, that Blakemore (2022) concluded to 1 m depth of ~ 2.1 × 10^30^ virions, based upon Bar-On et al.’s (2018: 55) summary they accepted had a 32-fold uncertainty. An indication of these uncertainties is from new soil virus data provided in 2023 (https://web.archive.org/web/20220301082457/https://www.soilviral.com/) having: “1 billion viruses g^-1^, that if calculated over the whole globe amounts to about 4.9 × 10^31^ soil viruses”. Doubled for terrain, this is ~ 1 × 10^32^ as a new upper value in a range, now of 10^31^–10^32^VLPs. A mean value of around 5 × 10^31^ global total virions on Earth is then a reasonable compromise, which why this value is quoted in the Abstract above.

Virus to Microbe/Bacteria Ratios (VtBs) of Virus-Like Particles (VLPs) interlink (as shown in Appendix 2) indicating likely ranges of both abundance and diversity acting as mutual cross-checks on relative abundance and diversity summaries. Wide ranging VtB estimations, pertinent for soil, mostly vary around 10:1 to 100:1. Emerson (2019) summarized how abundant and important viruses are in the Soil compared to in the Sea. A plausible summary is that viruses are most abundant in Soil and at least ten times, but often ≤ 100 times (or more?), as rich as the Bacteria, their primary hosts, in terms of both abundance and biodiversity.

Conversely, a few studies show a VtB ratio around 1:1 suggesting both be raised to 10^26^ species? From Blakemore (2023), since both global and Soil alone bacterial biodiversity are in the order of 2 × 10^24^, then virus diversity may range from at least as many up to 10^25^–10^26^ total Soil viral species.

Meanwhile, Anthony et al. (2023) in a Supplementary file had an intermediate value of 1,000 “Phage” species per bacterial species. They said: “Using the upper estimate of bacterial diversity (3.7 × 10^9^) and a ratio of 1000:1, we predicted the upper and lower ranges of viral diversity.” Despite this, they appear not to have applied it to their table 1 having just 3.7 × 10^11^ global “Phages” rather than 3.7 × 10^12^ species as they intimated. This again indicates their report needs a through review.

### ﻿Review of Soil abundance enumerations

An upper diversity “Phage” value in Anthony et al. (2023: table 1) of 3.7 × 10^11^ species is well below current estimates about 10^26^ viral varieties found mainly in soils. However, viruses are excluded from strict biodiversity assays by failing to conform as free-living and independent entities according to most definitions of the entities of Life with all their attributes and, often mutual, relationships.

Prior to 2022, an oft-repeated claim that soils support 25% of global biota was seemingly attributable to Decaëns et al. (2006: figs 1, 2) that had: “A rapid survey of invertebrate and vertebrate groups reveals that at least 1/4 [i.e., 25%] of described living species are strictly soil or litter dwellers, the main part of which is insects and arachnids (Fig. 1)”. [Fig. 1]. Note that key Soil microbes and fungi are entirely ignored. Since those authors’ data had total described species numbering ~ 1,500,000, their soils would presumably total just 375,000 species (they show with an unrealistically low < 5% Bacteria, viruses, and Fungi within this total, or ~ 18,750 microbial taxa?). Of ~ 360,000 soil animals in Decaëns et al. (2006: fig. 2), only 1% “Annelida” is shown, presumably 3,600 earthworm species, a wide underestimation, approximately half the true count of described species as known at that time.

Because Anthony et al. (2023) overlooked key studies (not least by Benton 2001; Williamson et al. 2017; Bickel and Or 2020; Blakemore 2018b, 2022, and Zhao et al. 2022) also ignoring GBIF (2016), they implied Decaëns et al. (2006) was the only previous work on soil biodiversity. Thus, Anthony et al. (2023) improperly conceded that, rather than 25% as claimed by soil “experts”, soils held 59% (stated as: “an average of 58.5% of life inhabits soil” and “considering most life on Earth together, the average proportion of species in soil across all three estimates (lower, central, and upper) is 58.5 ± 14.7%, excluding phage [sic]”), i.e., with a range of 44–74% of global biodiversity. This conclusion is nonetheless unsupported in their table 1 data with Earth’s 1.01 × 10^11^ and Soil’s 1.04 × 10^10^ of species that is ~ 10% (as in Table 3), mainly composed of “Phages”, which their figures show total 1 × 10^11^ species with 9.9 × 10^9^ (or implausibly just 9.9% of viruses!) in their soils.

“Phages”, if excluded from their totals, give Earth and Soil taxa values of non-Phage biota of 1 × 10^9^ and 0.5 × 10^9^, respectively, or with ~ 50% biota in soil. This value, of 500 million soil species, is orders of magnitude lower than values of 10^11^ soil microbes (mainly Bacteria) reported by Zhao et al. (2022), ≤ 10^23^ in Fishman and Lennon (2022), and 2.1 × 10^24^ taxa (almost all Bacteria) in Blakemore (2022, 2023). These latter studies reasonably exclude viruses which are difficult to accommodate within most definitions of true living entities, as has already been remarked on and adhered to herein.

In summary, of their 1.04 × 10^10^ soil species, just 500 million would be non-Phages but, of these, seemingly 4.4 × 10^8^ are “Microbes” composed mainly of 4.3 × 10^8^ “Bacteria”. Subtracted from 5.0 × 10^8^ non-Phage soil species, implies there are ~ 0.7 × 10^8^ or 70 million non-Phage, non-Bacterial species anticipated in their mean soil taxa total. Discrepancy in their table is that this figure appears to be higher than Earth’s total 0.1 × 10^8^ or 10 million non-Phage, non-Bacterial species! Such issues indicate a need for self-correction quality controls, possibly acknowledged correction or retraction.

Restating conclusions as herein, Zhao et al. (2022) reasoned that “soil is the most microbiologically abundant (∼ 10^29^) and diverse (∼ 10^11^) environment on the Earth”, however, this data was updated in Blakemore (2022, 2023) to an abundance of 2.1 × 10^30^ cells and 2.1 × 10^24^ soil taxa both comparing poorly with Anthony et al.’s (2023) central value of just 1.04 × 10^10^ total soil taxa. Differing by a factor of two and an order of × 10^14^, or a hundred trillion times, this disparity needs remedy in properly directed Soil research as an urgent priority if a dedicated Soil Ecology Institute emerges.

Resolution of shortcomings continues, as Wiens (2023) pointed out: “Mora and colleagues estimated approximately 10,000 bacterial species (roughly the number of described species). They acknowledged that these projections were likely underestimates. Yet, prokaryotes may be a major driver of Earth’s overall species richness. Recent studies have estimated a staggering range of species numbers for bacteria, from low millions to hundreds of millions, to low trillions. All were based on extrapolations from molecular studies.” He continued: “Clearly, controversies about global biodiversity cannot be resolved without better resolving bacterial richness”. Accepting that this is still a young and growing area of research, I wholeheartedly concur, adding that Soil is foundational.

### ﻿Context of Soil species extinctions

As biodiversity estimates climb, actual on-the-ground species decline due to rapidly increasing extinctions, up to 100–1,000 × above expected rates from IPBES (2019: fig. SMP3) of: “background rate of 0.1–2 extinctions per million species per year”. However, IPBES lacks both “Context and Triage”, thereby losing credibility, appearing to give equal status to Land:Sea:Freshwater when in factual reality these respectively provide 99.9:0.1:0.0% to biodiversity (or to humanity's thriving). Extinction is a large, complex topic, but some key references are E.O. Wilson’s (1992) prediction from rain forests of 27,000 extinctions per year (74 per day) and IPBES (2019) reportedly having a rate ≤ 200 species lost per day, mainly on land, and mainly for larger, charismatic taxa rather than the 99% of lesser, understudied invertebrates (so true base rate may be 100 × higher at 20,000 per day?).

Albeit soil faunal lists grow exponentially, our soils are being subjected to severe and accelerating destruction from erosion, desertification, chemical poisoning, capping, and rapidly increasing soil acidity – a critical global issue that is mostly ignored (cf. Raza et al. 2021; Zamanian et al. 2021). Soil loss inevitably results in silent species loss, mostly of microbes that are most dominant in soils (as this report indicates), but also of more obvious soil macrobes (e.g., Veresoglou et al. 2015), and specifically of earthworms (Blakemore 2018a) that in this regard are also remarkably understudied.

In the context of soil losses, no wholly marine mammal, shark/ray, fish nor coral is confirmed extinct in the last 250 years (Vermeij 1993), and nary a polychaete marine worm either (https://recentlyextinctspecies.com/databases/annelids). Freshwater losses have occurred, but the biodiversity of this biome is relatively minor and these almost always relate to the surrounding soils. The next section measures the magnitude of macrobe losses, with terrestrial Gastropoda (e.g., slugs or snails) as a useful model for proportionate extrapolation to the specifics of earthworm extinctions.

### ﻿Earthworm extinction losses

An extinction website (https://web.archive.org/web/20230718152549/https://en.wikipedia.org/wiki/List_of_recently_extinct_invertebrates) catalogues just three Annelida (earthworms), one each from Tasmania, NZ and Japan (each surveyed, evaluated and reported by myself, as per Blakemore 2018a), against 25 better-studied Arachnids (spiders). For terrestrial Mollusca gastropods (snails and slugs) their link (https://web.archive.org/web/20240406171442/https://en.wikipedia.org/wiki/List_of_recently_extinct_molluscs) has a higher total of about 428 extinct taxa. Compared to earthworms, some confounding factors are approximately an equal number of molluscs are marine or aquatic (although no wholly marine snail, nor worm, is confirmed as extinct in the last 250 years since Linnaeus’ Volume 1), while only a few earthworms are littoral or aquatic (~ 60 as remarked on earlier), thus land-based taxa approximations may be reasonably commensurate. Published extinction reports are presumably verifiable, whereas true extinction totals may be much higher since only a proportion of existing species are known, fewer evalutated. For earthworms, ~ 7,000 species are described with 30,000–35,000 total taxa expected; this corresponds well with terrestrial gastropods having a higher proportion of ~ 24,000 known species, but estimated total also around 35,000 species (Barker 2001).

Although gastropods as mostly superficial feeders are provisionally excluded from some soil fauna lists, they are like earthworms in two respects: They are wingless, thus are often highly endemic, plus the predicted total numbers of their taxa are on par. This is important because the better known and researched molluscs have published extinctions of ~ 400 species which may reasonably be applied to earthworms if their researchers had the same level of support as do Malacologists. Seemingly, due to such research disparities, ~ 42% of all studied and reported animal extinctions have occurred within this popular gastropod group (Lydeard et al. 2004). Economic arguments that molluscs attack plants are nullified by primal and proven enhancement of vegetation or crops due to earthworm activities.

How supportable is a > 400 earthworm species extinct estimate? Régnier et al. (2015) said: “Using data on terrestrial invertebrates, this study estimates that we may already have lost 7% of the [described living] species on Earth and that the biodiversity crisis is real.” And using this datum, Cardoso et al. (2020) stated: “However, it is likely that insect extinctions since the industrial era are around 5 to 10%, i.e. 250,000 to 500,000 species, based on estimates of 7% extinctions for land snails (Régnier et al. 2015). In total at least one million species are facing extinction in the coming decades, half of them being insects (IPBES, 2019).” Thus, for all ~ 7,000 currently described megadriles, a 7% loss would be ~ 490 species extinct. Q.E.D. Similar loss extrapolated to all > 30,000 of likely total megadrile earthworms (in the unlikely event anyone attempts to describe them all), would be > 2,100 extinct earthworms. Fixing the issue of potential losses of such an essential soil fauna, as was highlighted in a meta-analysis of organic farms by Blakemore (2018a), should be a major priority. A subsequent study from birdwatchers in the UK, while ignoring this global meta-analysis study, yet independently and subsequently came to a similar conclusion (Barnes et al. 2023).

As already noted for terrestrial invertebrates, Régnier et al. (2015) estimated critical 7% species loss while Cowie et al. (2022) had 7.5–13% loss, but the status of most taxa remains unclear. Isbell et al. (2022) regarded ~ 30% terrestrial invertebrates either threatened or extinct, which is similar to ~ 30% threatened or extinct rates in IUCN’s “Redlist” of earthworms of Japan and NZ/Australia compiled by the author in 2018. Yet most of the earthworm species in these reports were DD: “data deficient”.

### ﻿Microbial extinction losses

Although the IPBES (2019) report barely considered microbes nor “non-charismatic” invertebrates, they did note: “around 9 per cent of the world’s estimated 5.9 million terrestrial species – more than 500,000 species – have insufficient habitat for long-term survival, and are committed to extinction, many within decades, unless their habitats are restored.” Yet, their rate estimate of ≤ 200 non-microbe species lost per day, is mainly on land and mainly due to often irreversible Land-Use-Change (LUC for agriculture and/or pasture). If the massive new biodiversity estimates herein have similar and proportional rates, this may increase many-fold for the > 99.9% soil microbes in 10^24^ taxa. Proportionately, a 7% rate of invertebrate loss (noted above) would equate to 2 million microbes per day, or ~ 23 taxa lost per second! This critical issue as alluded to in the Abstract was reported here: http://vermecology.wordpress.com/2021/06/20/tol/ and requires further investigation.

An example for microbes is *Streptomycesavermitilis* (ex Burg et al., 1979) initially found only once in a soil sample collected in 1977 near a golf course at Ito, Shizuoka-ken, Japan. From this single species the Nobel-prized pharmaceutical Avermectins were derived. Just as the loss of the soil biome should be of concern for productivity and natural remedies, increasingly it is being recognized that dysbiosis of the human (or other animal) gut or superficial (skin) biome is also related to good health. This human health issue is outside the current study remit but closely relates to healthy soils.

Regarding microbe extirpations on farms, Blakemore (2018a) also noted microbial declines under artificial compared to organic fertilizers at Rothamstead, UK by -50%, likely from the onset of their chemical farming schemes. A similar loss of -50% Bacteria and fungi in chemical compared to organic husbandry was reported from farms in the Philippines (Blakemore 2017: table 5). A meta-analysis by Lori et al. (2017) obtained similar findings and came to similar conclusions on soil loss.

Bacterial (and lesser fungal) richness relates to soil carbon, and its reduction due to land use (poor farming) and climate change could cause dramatic shifts in the microbial diversity (Bastida et al. 2021). This is tenuously supported by a recent paper (Kačergius et al. 2023) at the Lithuanian Institute of Agriculture on organic, sustainable, and intensive-chemical farming systems that found: “20 years ago, when analyzing soil samples from the same agricultural fields, colonies of culturable bacteria and fungi were grown and up to 1–5 × 10^6^ CFU of organotrophic bacteria were counted, up to 1–2 × 10^7^ nitrifying bacteria. In 2022, we counted up to 1–4 × 10^5^ CFU during culturable bacterial colony counts, which is quite different than 20 years ago.” Although there are problems with this study (e.g., having to use “culturable” counts for comparison, and a highly acidic “pine old-growth forest” control), this relative decline, if truly representative, shows a 10–100 times fall in microbes in just 20 years. Losing a few species per year from just one site if applicable to farmlands globally could be significant. Were this trend more widely manifest it would be a major concern for anyone, not just Soil Ecologists, organic farmers, or policy makers. Confirmatory research is clearly required.

Recently, Thaler (2021) cogently noted: “Darwin’s “tangled bank” of interdependent organisms may be composed mostly of other microbes. There is the likelihood that as some classes of microbes become extinct, others evolve and diversify. Lack of insight into the dynamics of evolution of microbial biodiversity is arguably the single most profound and consequential unknown with regard to human knowledge of the biosphere”. In light of the current work few could now disagree with this.

## ﻿Summary, conclusions, and future directions for Soil loss remedy

Shortcomings in Decaëns et al. (2006) soil biodiversity summary as (shown in Fig. 1) are mainly that it only reports intensity of study, not estimated totals, and mostly ignores microbes that have since become paramount. Other flaws in its premise are that since around two million species had already been described at that time (MEA 2005: Chapter 4), then their 23% in 360,000 species would likely have been closer to ~ 18%. Conversely, if their > 23% in Plants, Fungi, Bacteria, and viruses that are all mostly found in soil were added to their 23% total, the soil proportion is doubled, being raised to > 46%, albeit only ~ 1% of soil organisms are known (FAO 2020). Hence, would a likely new total for their study if 100 × and of around~ 38 million species not already have a 99% majority in soils?

Moreover, it appears that estimated soil fungi alone supported as many as their claimed global total of 1.5 million species: “The estimated global fungal diversity has changed dramatically from 100, 000 in the 1940s to 1.5 million in the early 2000s, then 2.8 to 3.8 million in the 2017s, and currently 2.5 million species as the best estimate. However, 155,000 species are currently known; thus, many species are still undescribed and waiting for their discoveries” from https://mycokeys.pensoft.net/topical_collection/254/. These known fungi should also be in soil total.

Anthony et al. (2023) claimed their 1.04 × 10^10^ soil species estimate as “approximately two times greater soil biodiversity than previous estimates” but it was considerably less than Zhao et al. (2022: fig. 3A) already with 10^11^ soil microbial OTUs that was revised upwards to 2.1 × 10^24^ species by Blakemore (2022). Both prior studies surpass their subsequent 2023 findings indicating a need either for rebuttal or for a thoroughly refined restatement of both local and global soil biotic enumerations.

This review of vital Soil Biota aimed to clarify its true scope while indicating key areas in need of understanding. The vast array of faunal, floral, fungal, and microbial groups and their roles are mostly unexplored and open for investigation, emphasizing an urgent need to establish a Soil Ecology Institute. Until this is fully realized, in the interim, myriad Aquatic or Atmospheric facilities abound, although the naturally depauperate Ocean and void Space will mostly remain intact regardless as they do not erode, neither do they flood nor burn. Ocean issues are solved in Soil. Due to the most pressing problem of topsoil erosion and irreversible extinction losses, a major shift should be realizing the overwhelming importance and fragility of our precious Soil. The need for proportionate fund reallocation (hence no extra costs involved) to support urgent and directed soils research – under the principles of true Context with systematic Triage – to benefit all Life on Earth.

A supporting homage to our origins and reliance on Microbes in Soils is a diagrammatic Tree-of-Life, as alluded to in the Abstract and Introduction, showing common microbial ancestry origins and prehistoric extinction events – https://web.archive.org/web/20240705043415/http://evogeneao.s3.amazonaws.com/images/tree_of_life/tree-of-life_2000.png. The author notes that this is a phylogenetic tree not reflecting biodiversity.

This Tree-of_Life is particularly poignant with regards to a mostly mysterious soil virome as expounded by Paez-Espino et al. (2016). Williamson et al. (2017) discuss similar issues, coming to a simple conclusion: “Soils remain the most poorly understood ecosystems on Earth. At the same time, viruses represent the largest pool of untapped genetic diversity and unexplored sequence space on the planet. In this regard, the soil virome comprises an unknown quantity within an unexplored territory: a vast new frontier, ripe with opportunities for discovery.” The current report is not alone in realizing such magnitudes, nor in urging for more support for Soil Eco-taxonomic restoration in order to boldly explore this vast new frontier lying in wait directly beneath our feet, while it still exists.

While focusing on fundamental soil microbiome, it is important to note this is enhanced by activities of a literal ground-breaking master of its domain as manifest in Darwin’s “humble earthworm”.

Promoting earthworm activity, as advocated by Blakemore (2018a, 2022, 2023), increases plant growth and provides microhabitats for soil fauna and flora, viz.: “microbes increase during digestion and after gut passage in their fresh castings by up to × 1,000 (Lee 1985: 27, 206) further enriching soils.” Presumably the viral abundance is also increased by a multi-fold magnitude due to such actions. Thus, a simple solution to soil degradation is to attempt, in any way and at all times, to preserve and enhance earthworm populations that are more accessible than microbes. As Bill Mollison, co-founder of Permaculture and author of their Designers Manual (Mollison 1988) said: “There is one, and only one solution, and we almost have no time to try it. We must turn all our resources to repairing the natural World, and train all our young people to help. They want to; we need to give them this last chance to create forests, soils, clean waters, clean energies, secure communities, stable regions, and to know how to do it from hands-on experience.” (https://web.archive.org/web/20240719045254/https://www.azquotes.com/quote/873849).

That the Soil hosts > 99.9% of global diversity now requires a major “Sea change” in attitudes and funding to recognize its true scope. This should spur formation of at least one dedicated Soil Ecology Institute (for both natural and managed lands) tasked to catalogue, research and reverse mass degradation of our planet’s most crucial, yet most neglected ecosystem – that of the Soil Realm.

## ﻿Additional information

### Author ORCIDs

Robert J. Blakemore https://orcid.org/0000-0001-9797-8328

### Data availability

All of the data that support the findings of this study are available in the main text.

## ﻿Appendix 1

Biomass of earthworms and microbes, as major soil organisms in the various biomes, are compared to minor microdriles (viz. Enchytraeid potworms that were given inordinate importance by Anthony et al. (2023)). These data are presented in Table A1:

**Table A1.** Biome Carbon biomass of Enchytraeids, Earthworms and Microbes selected from Fierer et al. (2009: table 1– https://onlinelibrary.wiley.com/doi/epdf/10.1111/j.1461-0248.2009.01360.x) in g/m^2^ C, with Biome areas from Whitman et al. (1998: table 2 – http://rpdata.caltech.edu/courses/aph161/Handouts/whitman98.pdf) in Gigahectares (Gha).

Biome	Enchytraeid (g/m^2^ C)	Earthworm (g/m^2^ C)	Microbe (g/m^2^ C)	Biome (Gha)	Total Enchy. (Gt C)	Total E/worm (Gt C)	Total Microbe (Gt C)
Boreal forest	0.32	0.3	57	1.2	0.04	0.03	6.84
Desert	0	0	43	1.8	0.00	0.00	7.74
Temp. conif.	0.80	1.2	175	0.5	0.04	0.06	8.75
Temp. decid.	0.64	2.0	116	0.7	0.04	0.14	8.12
Temp. grass	0.31	3.8	131	0.9	0.03	0.34	11.79
Tropical forest*	0.10	4.9	203	2.5	0.03	1.23	50.75
Tundra	0.99	1.4	136	0.8	0.08	0.11	10.88
**TOTAL**				**8.4**	**0.26**	**1.9**	**104.8**
**Terrain × 2****				**16.8**	**0.52**	**3.8**	**209.6**

*Tropical forest Enchytraeid data infilled from (https://edepot.wur.nl/202864 1991: table 2.2 with 0.02–0.20 g dry wt. thus < 0.1 g C similar to FW (fresh weight) data extracted from https://soil-organisms.org/index.php/SO/article/view/155 2021:tabs 4, 5). Note that earthworms are usually the most important elements of Tropical forests biota, contrary to some misguided reports, as clearly explained by practical Soil Ecologist fieldworkers, e.g., Gijsman (1991). Enchytraeids have ~ 700 known species compared to ~ 7,000 described earthworms, or × 10, and biomass of earthworms is × 7 too. **Terrain doubling from Blakemore (2018b) allows for landscape’s coarse terrain progressively overlain by finer layers of microtopography and soil rugosity, less so in bogs.

Earthworm biomass at 3.8 Gt C and Microbes at 209.6 Gt C are slightly higher than 2.3–3.6 Gt C and 200 Gt C, respectively, as estimated by Blakemore (2023: table 2) confirming importance of both groups to Soil Ecology.

This new earthworm value of 3.8 Gt C may be doubled to ~ 7.6 Gt for dry biomass which is substantially higher than the 0.9 Gt (and thus 0.45 Gt C?) as lately reported in Miller et al. (2024). Their paper overlooked 4.5 Gt dry and 2.25 Gt C data in Blakemore (2017 -https://vermecology.wordpress.com/2017/02/12/nature-article-to-commemorate-charles-darwins-birthday-on-12th-feb/) independently extrapolated from the extensive works compiled by the leading earthworm ecologist and taxonomist, my PhD assessor and mentor, Dr Ken Lee (1985).

## ﻿Appendix 2

**Virus-Like Particle (VLP) counts (Global and in Soil alone)**

All viral estimates in Anthony et al. (2023: table 1) had speculative uncertainty marked “?”. Indeed, Williamson et al. (2017) found soil viral diversity severely underestimated and under-sampled, albeit their measures of viral richness were much higher for soils than for aquatic ecosystems. Many soil virus reports show ≤ 10^10^ –10^11^ virions per gram of soil and a global best-estimate tally was of > 10^31^ virus-like-particles (VLP) that are infecting microbial populations at any one time (Mushegian 2020 originally cited from Hendrix et al. 1999).

If 10^10^ –10^11^ virions per g occur, this is 10^16^ –10^17^ per tonne of soil. Further, if there exist 2.1 × 10^14^ t topsoil to 1 m depth (from Blakemore 2022), then a range is 2.1 × 10^30^–10^31^ virions (with a median value ~ 1.5 × 10^31^).

This total is approximately the same as that calculated by other authors, e.g., Mushegian (2020) or Cobián-Güemes et al. (2016) of between 10^31^ and 4.8 × 10^31^ viral phages. Comparatively, Suttle (2005) extrapolated counts of Marine viruses from local samples to the entire World, arriving at an estimate of 4 × 10^30^ virus particles in well-mixed oceanic waters (i.e., ~ 10%). Mushegian (2020: table 1) arrived at a similar estimate to this at 2 × 10^30^ virions in the Ocean, i.e., a range of just 2–4% of Ocean virions in a global total of ~ 10^31^ virions.

Previous range of Ocean virus proportions is then just 2–10% of global totals with much of the remainder (90–98%) in Soil. True Soil counts are variable, as shown below, further reducing the proportional Ocean values.

While initial estimates of virus abundance in Soil ranged from 10^7^ to 10^9^ virus like particles (VLP) per gram of dry soil (Williamson et al. 2005, 2017) with a mean ~ 10^8^, Pratama et al. (2020) found a higher mean of 10^9^VLP per g from a range of catalogued soils and Graham et al. (2023) had 10^7^ to 10^10^ viruses per g of soil. This agrees with Jansson (2023) for different soil types at 10^8^–10^10^VLP per g dry soil (mean also 10^9^), but she noted the true number may be higher than that obtained by microscopy because many soil viruses are intracellular and not able to be imaged separately. Kannoly et al. (2022) shows ≤ 1,430 plaque-forming-units (PFU) per lysogenic bacterial cell-burst (a so-called “burst size” of viral particles). Thus, an exponential order or two is easily added, possibly to allow reasonable estimates of around 10^10^–10^11^VLP per g of dry soil?

Especially relevant, a study by Cobián-Güemes et al. (2016) estimated 4.8 × 10^31^VLPs on Earth but comprising an unrealistic minimum of 257,698 different viral genotypes (sic). They quoted reports with only 3.9 × 10^6^ ≤ 2 × 10^9^ total varieties (or one variety per 10^22^–10^25^VLP), which seems a wide underestimation compared to other studies. Their VtB ratios (in their table 1) were skewed by a low “Human associated” ratio of just 0.1 and a high “Other host-associated” of 25, to give a median ratio for all their biomes of 12. Their Soil VtB was 19.1. Here revised microbial counts in Blakemore (2022, 2023) give a global total of > 5.1 × 10^31^VLPs with ~ 4.1 × 10^31^ (~ 80%) virions in soils (to full depth?) and a Soil alone VtB near ~ 20:1, as in Table A2:

**Table A2.** Virus-Like Particles (VLP) from Microbes/Bacteria (VtB) ratios modified after Cobián-Güemes et al. (2016: table 1) and Microbes abundances from (Blakemore 2022, 2023 cf. those given in Table 2 of main text above).

BIOME	Microbes/Biome × 10^28^	VtB ratio	VLP/Biome × 10^31^	Microbe %	Virus %
Marine	12	12.76	0.15	4.0	3.0
Freshwater	0.02	14	0.00	0.0	0.0
Sub-Ocean	40	11	0.44	13.2	8.6
Sub-Terrestrial	40	11	0.44	13.2	8.6
Soil *	210	19.5 (~ 20)	4.10	69.5	79.8 (~ 80)
TOTAL	302.0	(Mean 11.4)	5.13	100.0%	100.0%

*Their soil microbe value was 2.50 (truly 2.556) × 10^29^ whereas Blakemore (2022, 2023: table 3) had 210 × 10^28^ (cf. Table 2). Data was based upon Whitman et al. (1998) for Prokaryote cells, as revised by Blakemore (2022) and corrected (as detailed in text), with combined Sub-Ocean and Sub-Terrestrial values averaged out.

Whitman et al.’s (1998: table 5) 3.6 and 2.5 × 10^30^ cells in Oceanic or Terrestrial sub-surfaces were downgraded by Kallmeyer et al. (2012), Parkes et al. (2014), Magnabosco et al. (2018) and Hoshino et al. (2020) to just 3–5 and 2–6 × 10^29^, or ~ 4 × 10^29^ each, and with sub-surface biomass of 4 and 23–31 Gt C, respectively. Bar-On et al. (2018: supp: 62) for Marine, Sub-Ocean, and Sub-Soil, had 1.2 × 10^29^, 4 × 10^29^ and 20 × 10^29^ cells, respectively. For Soil, their Prokaryote total was ≈ 3 × 10^29^ cells, similar to the values presented herein.

The mean VtB ratio 11.4 is approximately the same as Cobián-Güemes et al. (2016: table 1) median VtB of 12, both above Bergh et al. (1989) ~ 10:1 aquatic VtB. If 11.4 is applied to 302 × 10^28^ Microbes a total is ~ 5 × 10^31^VLPs. However, Soil alone VtB is double at ~ 20:1 that, for 210 × 10^28^ Microbes, is ~ 4 × 10^31^VLPs (~ 80%). Thus, proportion of viruses in Soil from total viruses is ~ 80% as noted in the Abstract (cf. Ocean 2–10% noted above), with the remainder in the deep-subsurfaces.

Although most samples are superficial, often in just the top 5 or 10 cm of soils, viral activity persists throughout the soil profile, to at least 1 m depth according to Muscatt et al. (2023). Interestingly, these latter authors stated: “Viral contributions to soil ecology are largely unknown due to the extreme diversity of the soil virosphere. Despite variation in estimates of soil viral abundances (10^7^ to 10^10^ viruses per gram of soil), it is clear that soils are among the largest viral reservoirs on Earth. Early metagenomics investigations have revealed high genetic diversity in soil viruses, with putative impacts on global biogeochemistry. Still, less than 1% of publicly available viral metagenomic sequences are from soil, reflecting the lack of knowledge about soil viruses and their ecological roles”. Accordingly, as soil is so under-represented, Graham et al. (2023, 2024) argue that understanding the rôle of viruses in soil is most pressing of any of our ecological challenges.

**Virus to Bacteria (VtB) ratio abundances**

As already noted, Mushegian (2020) had an approximate 10-fold excess of phages over bacterial cells (as per Bergh et al. 1989), whereas Cobián-Güemes et al. (2016: table 1, fig. 1) median VtB was around 12:1 and in Soil alone ~ 20:1 (or 100:1 mean soil ratio in their figure which is an order higher that all other ratios). Applied to Table A2 totals, a 12:1 ratio for all ~ 3.78 × 10^30^ global microbe cells would be ~ 4.6 × 10^31^VLPs with 4.1 × 10^31^, or 89%, of viruses in Soil. But this too may be out by an order or more, not least to account for those active intracellular virus particles that are mostly overlooked in general surveys, or in the Soil’s VtB ratios.

Early on, Ashelford et al. (2003) averaged soil virus numbers at 1.5 × 10^8^ per g, which they said was equivalent to 4% of total bacteria population (of 3.6 × 10^9^ per g) giving a virus-to-bacterium ratio (VtB) in their soil of 0.04:1. Subsequently, other authors found much higher numbers, e.g. Cobián-Güemes et al. (2016: fig. 1) had mean Soil VtB ratio ~ 100:1 but selected a median value of ~ 20:1 as in their table 1 (cf. Table A2).

Cao et al. (2022) reported highly variable virus-to-bacteria ratios (VtB) in soils as ranging from 0.001 to 8,200 (six orders of magnitude!) although their study found abundance of virus-like particles (VLPs) ranged from 2.0 × 10^7^ to 1.0 × 10^10^ and microbial abundance ranged from 1.0 × 10^8^ to 8.2 × 10^8^ per gram of dry soil, to give a VtB ratio from ~ 0.1 to 98.3 (near three orders of magnitude range), settling around a median VtB ratio value of 10:1 compliant with Cobián-Güemes et al. (2016: table 1, fig. 1) but including depauperate aquatic biomes. Wide ranging (VTM/VBR = VtB) estimations, pertinent for soil, mostly vary ~ 10:1 to 100:1 which is interesting as this complies with a virus species to host species range as assumed by Koonin et al. (2023).

**VtB ratios (= VTM/VBR) for species diversity**

Although an answer is complex, a preliminary estimate in Fierer et al. (2007: table 3) had bacterial OTUs of 10^3–6^ (median ~ 5 × 10^4^) while viral vOTUs ranged 10^3–8^ (median 10^6^) thus, extrapolating data, soil viruses may appear 10–100 times more diverse than Bacteria as a rough indication of mutual biodiversity crosscheck. As just noted, Koonin et al. (2023) conservative range estimate was 10:1 to 100:1 for their host species ratio. Muscatt et al. (2023) determined: “overall vOTU per host ratio was 0.42 (median = 0) [sic], reflecting the predominance of unique host associations for individual vOTUs”. This suggests viral diversity is commensurate with Bacteria/Archaea diversity (vOTU:bOTU) and vice versa. So, for 2.1 × 10^24^ soil microbe species, viral diversity would be at least (2.1 × 0.42 = 0.88) or around 0.88 × 10^23^ vOTUs. Viral richness (~ 10^23^ per 10^31–32^ virions) would then be ~ 1 unique ‘variety’ for each 10^8–9^ virions, or roughly two or three orders lower than bacterial richness which, as noted, is around one bacterial taxon per million cells. Q.E.D.

Conversely, Roux and Emerson (2022) quote: “estimates of soil viral richness suggested the presence of 1000 to 1 000 000 genotypes per sample” and samples were traced as ~ 200 g wet soil, say ~ 100 g dry, to give around 10–10,000 soil genotypes per gramme (or around 10^3^ in a mean of 10^9^ virus particles per g which is also ~ 1 vOTU per million virion cells as with bacterial estimates). Thus, both are at a mutual 1:1 ratio. Recently, Graham et al. (2024) quoted 10^7^–10^10^ viruses per gramme of soil showing soils as the largest viral reservoirs on Earth and they reported averages of 40.01 (range 1–2,124), 35.48 (range 1–1,651) and 24.91 (range 1–896) unique viral clusters per soil sample (presumably 1 g?) at the species, genus, and family levels. This suggests four viral species for each one million, up to a billion soil virions, a bit higher than for Bacteria.

A likely summary is that viruses are most abundant in soils and at least ten or 100 times as rich as the Bacteria, their primary hosts. From Blakemore (2023), as both Global and Soil alone bacterial biodiversity are in the order of 2 × 10^24^, then virus diversity may range from at least as many, ≤ 10^25^–10^26^ viral species in total.

Alternatively, as ~ 10^31^ viruses are known, may soil Bacteria reasonably range 10^29^–10^30^ species?

Support is found in Kuzyakov and Mason-Jones (2018), viz.: “The total number of viruses (including intracellular viruses inside bacteria) is probably 1–2 orders of magnitude higher than the bacterial populations” From this we may again conclude in circular argument that Bacteria are 1–2 orders less in terms of both cells and species.

Finally, we may concur with Williamson et al. (2017): “To understand the soil virome, much work remains.”
